# Conceptualizing suffering and pain

**DOI:** 10.1186/s13010-017-0049-5

**Published:** 2017-09-29

**Authors:** Noelia Bueno-Gómez

**Affiliations:** Department of Philosophy and Research Center Medical Humanities, University of Innsbruck, Innrain 52d, A6020, Innsbruck, Austria

**Keywords:** Pain, Suffering, Mind-body problem, Medicine, Phenomenology

## Abstract

**Background:**

This article aims to contribute to a better conceptualization of pain and suffering by providing non-essential and non-naturalistic definitions of both phenomena. Contributions of classical evidence-based medicine, the humanistic turn in medicine, as well as the phenomenology and narrative theories of suffering and pain, together with certain conceptions of the person beyond them (the mind-body dichotomy, Cassel’s idea of persons as “intact beings”) are critically discussed with such purpose.

**Methods:**

A philosophical methodology is used, based on the review of existent literature on the topic and the argumentation in favor of what are found as better definitions of suffering and pain.

**Results:**

Pain can be described in neurological terms but cognitive awareness, interpretation, behavioral dispositions, as well as cultural and educational factors have a decisive influence on pain perception. Suffering is proposed to be defined as an unpleasant or even anguishing experience, severely affecting a person at a psychophysical and existential level. Pain and suffering are considered unpleasant. However, the provided definitions neither include the idea that pain and suffering can attack and even destroy the self nor the idea that they can constructively expand the self; both perspectives can b e equally useful for managing pain and suffering, but they are not defining features of the same. Including the existential dimension in the definition of suffering highlights the relevance of suffering in life and its effect on one’s own attachment to the world (including personal management, or the cultural and social influences which shape it). An understanding of pain and suffering life experiences is proposed, meaning that they are considered aspects of a person’s life, and the self is the ever-changing sum of these (and other) experiences.

**Conclusions:**

The provided definitions will be useful to the identification of pain and suffering, to the discussion of how to relieve them, and to a better understanding of how they are expressed and experienced. They lay the groundwork for further research in all these areas, with the twofold aim of a) avoiding epistemological mistakes and moral injustices, and b) highlighting the limitations of medicine in the treatment of suffering and pain.

## Introduction

This article aims to contribute to a better conceptualization of pain and suffering by providing non-essential and non-naturalistic definitions of both phenomena. Such definitions will be useful to the identification of pain and suffering, to the discussion of how to relieve them, and to a better understanding of how they are expressed and experienced. The provided definitions lay the groundwork for further research in all these areas, with the aim of avoiding epistemological mistakes and moral injustices such as the exclusion of certain experiences from the definition of suffering. Definitions are not inconsequential, since the way in which we define concepts has epistemological, ontological and practical dimensions.

Classical evidence-based medicine understands pain from a naturalistic point of view, and persons as beings are divided into two different entities: the body and the mind. Even if this perspective has led to great success in the relief of pain, certain problems have remained partially or entirely unresolved and/or unexplained, for instance the placebo effect, chronic pain and non-somatic pain. Moreover, classical evidence-based medicine has been increasingly criticized from the second half of the twentieth century onwards. This paper will begin by explaining the conceptions of pain and person used by evidence-based classical medicine and their Cartesian roots, followed by a critical discussion of the contributions made by the humanistic turn (represented by Cassell), and finally, the phenomenology and narrative conceptions of the self and the person.

An alternative to the mind/body dichotomy is assumed, consisting of an understanding of persons as psychophysical, socioculturally situated beings. Both pain and suffering have bodily, psychological and sociocultural dimensions. Pain (like pleasure) has been defined as a process resulting from a somatosensory perception, subsequently present in the brain as a mental image and followed by an unpleasant emotion as well as changes in the body [1], but such a process cannot be described exclusively in these neurological terms. Cognitive awareness [2], interpretation [3], behavioral dispositions [1], cultural [4] and educational factors [1] influence the perception of pain – for example, pain tolerance or the pain threshold.^1^ Suffering is proposed to be defined as an unpleasant or even anguishing experience which severely affects a person at a psychophysical and an existential level. Even when suffering is not caused by biological or observable circumstances (like the pain associated with tissue damage), it is an embodied experience which we cannot but feel in the rhythm of our hearts, the clenching of our stomachs, the sweat on our hands, our (in)ability to sleep, or the position of our shoulders, just to provide a few examples. Even if suffering does not originate from illness or pain, it can make us feel ill and can even cause us to develop various ailments. Pain can be a source of suffering, but it is not the only one. Social problems like poverty, social exclusion, forceful social inclusion (like peer pressure), forced displacement and uprooting; existential and personal problems like grief and stress; conditions like nausea, paresthesia, a non-painful illness, anxiety or fear can likewise be a cause of suffering. Although pain and suffering are unpleasant, they are not per se either destructive or constructive forces which tear down or build up the self. Rather, they are part of a person’s life, and the self is the result of various experiences *including* pain and suffering, which have an existential dimension inasmuch as they depend on the person’s attitude, resources for their management, as well as choices and commitments related to that person’s attachment to life and the world. Such personal options are influenced by social [5, 6] and cultural [7, 8] patterns.

## Background

### The mind/body dichotomy

Even if the “problem of consciousness” – “how consciousness arises from matter or, more cautiously, how it is related to matter” [9] – is far from a definitive solution, there is a generalized agreement in literature (particularly in sociology and the philosophy of medicine) regarding the need to question the traditional Cartesian distinction between the body and mind [1–3]. Kügler argues for the impossibility of conclusively solving the problem of consciousness, concluding that philosophy must continue working on this topic. However, such difficulties (or even impossibilities) may be due to the fact that we continue to use the classical concepts: We cannot resolve this dualism if we still think in dualistic terms. In order to reframe the mind/body problem, we need to think in terms of “embodying the mind” and “minding the body.”^2^

After questioning the mind/body dualism, the concepts of suffering and pain need to be reconsidered, even if a new conceptualization is indeed difficult [10, 11]. Simply put, it is no longer acceptable to consider pain only in physical and suffering only in psychological terms. The Cartesian distinction between *res cogitans* and *res extensa* is the driving force behind the whole structure of thinking in and the organization of medical sciences and psychology. Once we question this distinction, we need to reconsider this structure of thinking and organization, as well.

Questioning the distinction between the body and mind is not a new idea, despite its persistent prevalence in Western thought. The materialistic understanding of the mind (one of the alternatives to the mind/body dichotomy) can be traced as far back as the philosophy of Epicurus.^3^ In fact, there exists a whole alternative perspective, parallel to the Cartesian conception of the body and mind, developed by Spinoza and continued by Nietzsche and the American pragmatists (particularly William James), as pointed out by Johnson [10].

For Descartes, the body and mind are two different substances with a different ontological status: The body is like a mechanism that exists in time and space, it can be measured and so can its reactions and processes; however the mind lacks these spatial and temporal dimensions and can exist without a corresponding body. Accordingly, pain is something which occurs in the body and which can be described in terms of visible, physical, measurable damage (for example, tissue damage). In a period of increasing importance of the natural sciences, the Cartesian conceptualization of the *res extensa* presupposes a knowable world, organized according to certain natural laws [12]. It assumes that it is possible and desirable to intervene in the world scientifically to further the progress of humanity, which includes medicine, in particular. By using scientific methodology, it is considered possible to repair a body in the same way in which we can repair a machine (or an animal, inasmuch as Descartes considers animals part of the *res extensa*). Descartes himself is engaged in the enterprise of knowing the world in order to turn humans into “maîtres et possesseurs de la nature” (“masters and possessors of nature”) [12], proposing a scientific method and using it to improve living conditions. He trusts in human reason to the point of believing that progress in medicine will be able to relieve us of illness and even the weakness associated with old age, thus showing the first signs of an attitude which reaches its peak during the Enlightenment and declines (in a certain sense) in twentieth century, when the risks of scientific and technological intervention started to become apparent. The Cartesian perspective drove the development of clinical medicine as an empirical science based on evidence.

However, for Descartes, it was clear that our states of mind (“esprits” in the original French) depend on the “disposition of the organs of our body” [12]. Hence, medicine should contribute not only to the physical, but also to the spiritual and mental wellbeing, and ultimately result in “wiser” humans, both because medicine is able to provide scientific knowledge about human body (which constitutes a contribution to wisdom), and because medicine provides useful knowledge about the body which might allow humans to be free of illness and weakness, thus enabling them to develop and apply their intelligence to increase the knowledge of humanity. In short, it is not true that the body does not matter to Descartes, who was a rationalist but not an idealist, in the sense that he was not willing to risk his “corporeal” existence in order to defend his ideas (he preferred to accept rules and laws of his time that were incompatible with his own ideas in order to avoid imprisonment and other legal consequences, even though he supported the autonomy of reason). In this sense Cartesian dualism does not imply a dismissal of the body. Still, Descartes argues for the existence of an immortal soul which can stand on its own, without a body. Herein Damasio sees Descartes’ “mistake”: in the idea that the mind can exist or even operate independently of the body [1].

### The conceptualization of pain and suffering in classical evidence-based medicine

Pain and suffering cannot be treated exclusively in naturalistic, scientific terms, at least under a certain view of what science is. Medicine became a science at the end of the eighteenth century with the emergence of clinical, evidence-based medicine. In the context of such medicine, suffering and pain were dissociated from the context of a theodicy [13] and to be treated scientifically. Medicine started to be systematically organized in clinical environments, where patients could be observed and the symptoms and diseases compared and described as neutrally as possible: As explained by Foucault, the physician must distance himself from the diseased in order to learn the truth of the pathological fact [14]. Disease and pain started to be considered as being situated in bodies, since bodies and their processes came to be viewed in standardized, universalizeable terms. Knowing the medical, scientific truth about pain required both abstracting the body from the person, and the pathological fact from all normal bodily functions. These developments gave rise to the modern problematic approach to dealing with pain and suffering. According to Rey,

“At the dawn of the 19^th^ century, physicians were looking for a pure sign which would remove the ambiguities inherent in symptoms. They wished to find a sign, the meaning of which would be as certain as that provided by the lesion found at dissection. However, they were to be confronted not only with the multiple signs fundamental to pain, but also by that special exchange between physician and patient in which, whether consciously or not, the latter adopts a distinctive attitude in relating the details of his painful symptoms” [4].

The challenge of medicine based on observation, objective description of symptoms and diseases^4^ and experimentally proven treatments is dealing with a phenomenon like pain, which may or may not correlate to physical symptoms, whose relief may or may not be affected by the administration of certain drugs, but not always and not to the same degree, and which is definitely modulated by circumstances which are difficult or impossible to measure scientifically, like educational factors moral or religious beliefs, or personal attitudes. Pain is not a kind of spring, and bodies are much more than mere mechanisms, as phenomenologists have striven to show in the 20th century. Abstracting the “pathological fact” from the body and the body from the person facilitated a number of impressive results, treatments and medical progress. However, it proved to have its limitations too.

Pain has not been at the center of medical interest for the whole history of medicine. Of course, pain, like suffering, has always concerned medicine, but treating diseases in the search for healing and accumulating the necessary knowledge and expertise to do so more effectively in the future may be a better definition of the general goal of medicine in all times [4]. The Hippocratic moral maxim of “primum non nocere” has frequently been interpreted in this sense: To inflict pain (iatrogenic pain) can be considered “non nocere”, that is, not harmful, if it is done for the ultimate goal of curing the patient. In fact, the idea that greater pain can erase lesser pain is also of Hippocratic origin. This principle was particularly used during the nineteenth century by physicians who believed that pain can be useful for the purpose of healing [15]: The “moxa” procedure (direct moxibustion) consisted of placing a burning cone on the skin of a patient suffering from an ailment in order to infuse the body with external energy and stimulate the healing process. The pain resulting from the burn sore was seen as essential in swaying the body to combat the illness or pain the patient was suffering from in the first place [4]. We are usually willing to accept certain nuisances or even strong, painful secondary effects of medical treatments if we take them to enhance the recovery process or our quality of life. More questionable is the damage inflicted in order to prevent a more or less probable future disease, and an entirely different discussion concerns the damage inflicted in order to improve the knowledge of the discipline. In any case, the fact of the matter is that medical treatments and healing can – and usually do have – painful consequences, and they can cause suffering.

The attitude of trying to view the ills in the abstract in order to know the scientific “truth” of the pathological fact, and the empirical methodology, combined with the idea that healing is the ultimate goal of medicine, were precisely the focus of the criticism leveled against medicine, the new demands of patient and professional organizations, as well as the discipline of bioethics beginning in the 1960s. All these demands for a “more human” form of medicine were developed in a social context of alarm about the risks of techno-scientific progress and the general questioning of authority on many fronts [16, 17]. This criticism came to be known as the “humanistic turn” and it emerged from different fronts: the hospice movement [18], women’s rights movements which advocated a more active role of women in childbirth [19], Christian humanistic criticism against medicalization [20], bioethics and its criticism of medical paternalism [21], postmodern criticism of medicine [22], the “medical humanism” exemplified by Cassell’s work [3], and phenomenological as well as narrative approaches to the practices of medicine and the experiences of the patients, not to mention the contributions of the history, philosophy and sociology of medicine, which placed an emphasis on its fallibility and limitations, its historical and sociological dimensions, and, last but not least, its ontological assumptions. Due to this intense, yet unfinished debate and criticism, clinical medicine has begun to change, incorporating more or less parsimoniously any of the required reforms, while simultaneously increasing its techno-scientific dimension [23].

These theoretical critical approaches and the parallel social activism challenged the methods, goals and consequences of medicine in different ways. For example, the hospice movement is particularly relevant concerning the aforementioned predominance of the “healing goal” instead of the “palliative goal” of medicine. Cicely Saunders and Elisabeth Kübler-Ross pioneered this movement by emphasizing the necessity of taking care of patients even if their diseases are incurable. Displacing the goal of healing and situating “care” in itself as a focus of healthcare assistance involved increasing interest in the phenomena of pain and suffering in all their dimensions, as well as the research dedicated to improving and implementing analgesia.

All these critical approaches coincide in a demand for the resituation of the ill person in medical contexts. The patient should not be considered a “patient” anymore – a passive being patiently waiting for treatments and medical examinations. The modern patient expects to negotiate the medical decisions concerning them, because medical decisions are never strictly “scientific”, but also moral and/or political. For example, the decision to accept or reject a medical treatment in order to prevent a possible disease cannot be taken “objectively” because this is not a purely objective decision; it involves issues like the evaluation of the secondary effects of the treatment, the personal values and priorities of the affected person, or his/her ability to assume the risk. The scientific dimension of the decision is certainly only one among many. So the challenge mentioned previously still persists, since the physician is now required not to make an abstraction of the ill person, not to look at the body as if it were a mere mechanism to repair, not to take into account only somatic pain, but also to consider non-somatic pain, secondary effects of treatments, personal circumstances, etc. This situation requires the reconceptualization of pain and suffering, and a serious debate about the goals of medicine and its role in society.

## Results

### Cassell’s medical humanism

The work *The Nature of Suffering and the Goals of Medicine* was first published in 1982 and has had considerable influence on the ensuing debate regarding the medical conceptualization and management of suffering and pain. In fact, this debate has not yet ended [24–26]. This work can be classified among the theoretical works of the “humanistic turn” in medicine. Cassell criticizes clinical, evidence-based medicine, its dependence on Cartesian dualism, its conceptualization of pain and suffering, its management of them, as well as the goals of medicine. He criticizes exactly those characteristics of medicine which transformed it into a science in the first place, that is, the abstraction processes mentioned above, the fact that “doctors are trained to focus on diseases and to keep their similarities in mind, not their differences”, and that “the diagnostic methods are designed to see the same thing in each case of a disease” [3]. For him, the anachronistic division between body and non-body, and the focus on the cure of bodily disease, leads medicine to do things which cause the “patient as a person” to suffer. In other words, it not only treats pain inadequately (understanding and treating it only in relation to its measurable, observable and generalizable signs, in the context of a disease) but it also produces suffering, which persists undiagnosed and unrelieved, as is the case in the terminal phase of a chronic disease, which is progressively lengthened due to the availability of new treatments. In contrast, Cassell’s conceptualization of pain and suffering emphasizes their meaningful dimensions and the negative consequences of abstracting the pain from the person in pain. It takes into consideration that it is always an individual who feels pain or suffering, and that such experiences are modeled and strongly determined by personal assumptions, cultural patterns, cognitive activities and even religious beliefs.

Cassell defines pain not only as a sensation, but also “as an experience embedded in beliefs about causes and diseases and their consequences”, and suffering as “the state of severe distress associated with events that threaten the intactness of person”. Both pain and suffering are considered to have physical and psychological dimensions, and in this sense, it is true that Cassell avoids the classical association between pain and body, suffering and mind.^5^ His definition of pain is in line with the definition offered at the beginning of this article: Pain is a phenomenon which includes both nociception – “the mechanism involved in receiving painful stimuli” – and the subsequent attachment of meaning to such sensation. He recognizes the universality of nociception (“certain kinds of stimuli elicit the sensory response of nociception in every culture, now and forever”), but does not consider pain to be the same as nociception; for him, pain includes the meaning which the subjects ascribe to nociception, and such meaning changes from culture to culture, from person to person.

According to Cassell, suffering starts when “the sick person will believe that his or her intactness as a person is in danger”. So pain does not necessarily entail suffering, and suffering (a threat against the “intactness of a person”) can be caused by other experiences. Cassell proposes that medicine should be more sensitive to the person and the meanings he or she attributes to his or her pain/illness, and that it should specifically treat suffering, thus involving particular “subjective resources” like “feelings, intuition, and even the input of their senses” in order to deal with the suffering of patients. Other authors have also emphasized the importance of particular capacities such as sensitivity and empathy in a physician [27], developing an “affective mode of understanding” [25] in the context of trying to humanize medicine. But Cassell also thinks that it is possible to develop a methodology which is able to turn the subjective dimensions of pain and suffering into transmissible information that physicians can use in order to develop more holistic treatments (not only designed to cure a disease, but to palliate the suffering of the ill person). In this manner, the goals of medicine ought to be reformulated.

However, at least two problems arise from Cassell’s conceptualization of suffering. The first one is that his definition of suffering depends on a questionable understanding of the person and it is too restrictive. Defining suffering as a threat against the “intactness” of a person entails an assumption of what an “intact” person is. Cassell’s normative definition of “person” includes a number of dimensions like their perceived future, personality and character, body, past experiences and memories, cultural background, behavior, relations with others, a political dimension and a secret life [3]. This “intact” person would have developed a kind of equilibrium, or coherence and integrity, among all these dimensions.

Svenaeus [24] recognizes this difficulty inherent to Cassell’s proposal, the problem of thinking of “the person as a kind of whole” (or how it is possible to formulate a kind of integrity among all these dimensions), and offers an alternative: understanding life as a narrative and “stressing the experiential dimension, the holding together of states of consciousness making up the self”. However, the narrative explanations of the continuity of the self and life can be criticized, too. Although human beings have narrative experiences and dimensions, neither the selves nor life are completely and definitely unified by a single narrative. The stories we tell ourselves about our own experiences are certainly important resources which we use to relate to ourselves, to develop *our selves*. But such stories are not the only resource we use for such purposes. For example, we also engage in dialogue with our selves – the process of thinking has been defined as a kind of inner dialogue [28] – and a dialogue is not a story. Moreover, such inner stories are always pluralistic: They interpret our past experiences in the light of present interests or experiences. Hence we do not tell ourselves the same story about our past during our whole life, simply because our past changes every day as we gain new experiences which can easily modify the interpretations of previous experiences, and we need/want to understand our past differently according to our present and our prospects. Much more malleable and uncertain are our stories about the future: The future is unknown territory that slowly becomes present and then past, surprising us again and again.

In parallel, life is not “a narrative”, one single narrative from birth to death [29]. Different versions and interpretations about the life of a person are continuously written from different points of view; there is never a definitive history. Stories about life are always fragmentary, partial, and they cannot be told but from a certain perspective, depending on the intended emphasis. They do not guarantee the wholeness among our several dimensions.

Thus, the narrative explanation of the “wholeness” of the person does not support Cassell’s definition of “person”. Indeed, such a definition is a non-existent ideal which incorporates the idea that persons are transparent for themselves (they know themselves completely), coherent, able to design a kind of unique personal past and future story, and well balanced. This definition is far from being up to date regarding the contemporary theories of the self. Albrecht Wellmer [30] mentions two crucial contributions that contradict Cassell’s definition. Freudian psychoanalysis challenges the idea of an autonomous subject: Human beings do not always know exactly and completely what they want, what they do or why they do it, since they are influenced by psychological, social and power-relations forces. Wittgenstein and the philosophy of language challenge the idea that the subjects are the last authors and judges of what they say. Our meaningful expressions are not completely transparent to ourselves. Moreover, postmodern theories emphasize the contradictions among various social roles of the same person [31], our irrational dimension, our contingent nature and the fact that our actions are not predictable (even by ourselves). A person is never fully coherent, a person cannot be “intact” because touching and being touched is intrinsic to life. It may still be possible to define suffering as a threat to what a person considers to be his integrity at any given moment. However, this is an essential definition of suffering, which is too far-reaching and causes problems when trying to determine the boundaries of what is and is not suffering. Suffering can be experienced in different ways, not necessarily as a threat against one’s integrity, as I will show later. So this definition is unable to properly identify what is common to all experiences of suffering. Moreover, suffering has been seen and is often used to enhance identity (as in the case of the deliberate search for suffering, like self-inflicting pain, and other risky behavior). This stands in direct opposition to Cassell’s definition because seeking out suffering (or using non-deliberate suffering) is used to build or enhance identity, to affirm the self or to identify oneself with certain values like strength or courage.

The second problem of Cassell’s definition of suffering is discussed by Braude [25]: The experience of suffering may have a truly subjective element that cannot be explicitly communicated through language and “can and should never ultimately become an object, medical or otherwise”. Medicine can pay more attention to the aforementioned subjective, symbolic dimensions of suffering and pain, physicians can be trained to be more empathetic towards ill persons and more sensitive to their real needs. This “humanized medicine” provides a better management of pain and suffering, and it should reconsider its ultimate goals. However, the question remains whether suffering can really be treated solely by medicine and with purely scientific methods, considering this ultimately incommunicable dimension, the fact that not all kinds of suffering are related to pain or disease, and the existential dimension of suffering, which includes personal choices related to the attachment of the person to life and the world. Medicine does indeed have its limits.

### The phenomenological approach

The phenomenological conceptualization of suffering and pain offers an attractive alternative to dualistic theories and the mechanical understanding of the body.^6^ Contrary to the scientific approach, in which the body is seen from a third-person perspective, phenomenological proposals assume the perspective of the experience lived by a subject [32, 33]. This is a kind of first-person perspective that aims to be meaningful and relevant to others. A good phenomenological approach is not merely a subjective narrative of a personal experience, but is able to capture crucial elements of such an experience which are useful as meaningful resources for other persons trying to understand similar experiences.

A very good example of such a perspective can be found in Jean-Luc Nancy’s text *L’Intrus*, in which he aims to understand his own “lived experience” of heart transplantation, the associated severe medical treatments and their acute secondary effects, like lymphoma, philosophically and phenomenologically [34]. Nancy conceptualizes his experience not merely by telling his story, but by understanding it theoretically through the use of the concept of the “intrus” (intruder) and the idea of “intrusion” to understand the experience of receiving a new organ, its rejection by his immune system, of being treated “medically” (measured, tested, monitored), and finally the cancer and the subsequent treatments. His described strangeness of himself and his experience of liminality are far from unique, and his reflection about the moral consequences of organ transplantation and the increasing technological and scientific medical options all raise important points for further debate. In short, phenomenology is not merely subjective (although it incorporates personal experience) and good phenomenological approaches are powerful philosophical tools. Inasmuch as they are able to incorporate the first-person perspective, the “lived experience”, they possess a high potential for studying suffering and pain from a perspective which is not purely scientific or medical in nature.

With notions like “embodiment” and “living body” – the English translation of the German term “Leib”, in opposition to the “Körper” or “physical body” [11] – phenomenologists have contributed to “embodying the mind” by emphasizing the crucial role of the body in human experience and by assuming that we experience the world through our living bodies [32]. This assumption entails different consequences for the understanding of pain and suffering, such as the idea that if we are in pain or we suffer, we feel this displeasure in our bodies, thus influencing partially or totally how we experience the world. A transparent, silent or even an “absent” body [32] can become painfully present, so we experience the world from this painful perspective.^7^

Phenomenological approaches have contributed to “minding the body” too, as is the case with the phenomenological explanation of the “placebo effect”, one of the phenomena which challenge classical explanations of medical science. Frenkel [35] formulates this challenge as follows: “How could a private subjective expectancy associated with taking a placebo pill ever manifest as an observable, public change in the physiologic body?” The placebo effect particularly challenges the mind/body distinction and the consideration of the body as a mere “measurable object.” The explanation offered by Frenkel is convincing: The body itself is able to respond meaningfully to a demanding situation, since “we have a sentient body, capable of responding to the world without having to invoke any reflexive activity.” It is even possible to go one step further: If we conceive a person as a psycho-physical whole, it is not implausible to think of the body reacting in meaningful ways, that “a patient perceives affordances of healing in a particular situation and his body thus responds to the solicitation made upon it in the same way that our unreflective motor activity unfolds in the world.” Cultural, social and psychological factors are believed to affect the affordances (solicitations of response for a subject in a particular situation) of healing.

As already mentioned, Svenaeus [24] has combined phenomenological tendencies with narrative conceptions of personal identity in order to conceptualize pain and suffering. He puts together different definitions of suffering provided by other authors in an attempt to encapsulate “the whole of suffering.” However, uniting these different approaches to suffering does not guarantee a good definition of suffering, Instead, it guarantees a good overview of the studies or conceptualizations of suffering. A good definition should be general enough to include all instances of suffering. This does not mean that particular descriptions of cases of suffering are not useful or meaningful to other sufferers, scientists and simply persons interested in understand the phenomenon of suffering. To put it in other words, the alienation of the self described by Nancy can capture one essential dimension of one kind of suffering, but it does not define all kinds of suffering. Definitions of suffering as a threat against an “intact person”, as an alienation of the self, as an “alienated mood” or “unhomelike being in the world” [33] express different experiences of suffering, but these are not universal descriptions, so they are not good definitions. As Kleinman states, “It is important to avoid essentializing, naturalizing, or sentimentalizing suffering. There is no single way to suffer; there is no timeless or spaceless universal shape to suffering.” [7].

### Losing the self or finding the self?

As stated before, it is still a challenge for medicine to deal with these subjective, unmeasurable dimensions of suffering and pain – and, moreover, their possible “unshareability” [6], although there have been crucial contributions like the Gate Control Theory, which has been decisive in including both the physiological and the psychological dimensions of pain as intrinsic parts of the phenomenon. Still, pain and suffering do not only concern medicine, but also the social sciences and humanities, which contribute substantially to the clarification of their cultural, social and cognitive dimensions. If we attach importance to these dimensions in the experiences of pain and suffering, then we need to recognize the relevant role which said disciplines can play in making sense of them as well as in the provision of resources to relieve suffering. This ties back to the previous statement of medicine having its limits: There are types and dimensions of suffering whose management does not concern medicine (or at least, not exclusively). For instance, we cannot manage social problems that cause social suffering, like poverty, with medical resources. But as stated above, this does not mean that medicine cannot improve its management of pain and suffering: On the contrary, efforts to do so are already being made, even though a complete revolution will require truly overcoming the classical mind/body dichotomy.^8^ A real, coherent assumption of the person as a psychophysical instead of a dualistic being demands not only partial reforms in dealing with suffering and pain, but a total paradigm shift in the sense of Kuhn [36].^9^ In the meantime, interdisciplinary approaches are being put into practice; for example, the treatment of chronic pain in the long term now incorporates conductist therapies to manage its emotional and cognitive consequences [37, 38], or the treatment of non-somatic pain (for example, fibromyalgia) is now supported by psychotherapy [39].

The alienation (or even “loss”) of the self or the “unhomelike being in the world” can undoubtedly be consequences or expressions of suffering. Kathy Charmaz [40] describes the “loss of the self” in chronically ill persons and contributes to the understanding of suffering as not limited to a mere “physical discomfort.” In his recent, posthumous novel *Paris-Austerlitz*, the writer Rafael Chirbes describes the last phase of a man’s mortal illness in the following words:

“Rather, I had the impression that the man lying there wasting away became a stranger in both my eyes and his own – someone unknown to me, of course, but also to himself, and so Michel himself expressed it to me on days when he experienced a moment of lucidity. [...] Michel was being extinguished, fading just the same as each day of my visit, the dim light of the winter afternoon was fading in the frame of the hospital window.”^10^ [41].

Like Nancy, Michel cannot recognize himself anymore, and neither can his friend. For Svenaeus, suffering alienates us from our own body, from our engagements in the world with others, and from our life values [24]. “Alienating” means “making alien”, thus suffering is found to be equivalent to the feeling of being strangers to ourselves, to others, or to fitting into the world in an strange way – and it can impede us in living the lives we wanted. The alienation of the world can also be categorized as “unhomelike” in a way similar to Arendt’s concept: “Unhomelike being in the world” means that we exist in an uncomfortable way, in a strange, uneasy environment where we cannot rest or find our place [42].

These various contributions to understanding different experiences of suffering have not necessarily been proposed as essential definitions of suffering. For example, Charmaz’s work assumes a clearly situated perspective; she analyzes “a fundamental form of suffering” of chronically ill persons in America in the 1980s [40] However, there does exist a risk in taking such descriptions of suffering as universal, essential definitions, since doing so may have undesirable epistemological and moral consequences.

The idea of an “alienated self” presupposes the idea of a kind of “authentic self” with an “authentic life story”. Suffering can alienate us from our previous concerns and can even displace us into a state of liminality, where we do not feel at home in the world or in our bodies as we once used to. However, as stated previously, these are not definitive consequences of suffering, and persons are not static, unchangeable beings. Alongside the possible “loss of the self” exists the possibility of “reconstructing the self” (we were not our “definitive self” before “losing ourselves” due to suffering and we cannot recover something like a “definitive self”). Instead, we are the result of our experiences, including suffering and pain.

The proof that essentialist definitions of suffering do not hold is that two contradictory answers to the problems of pain and suffering can be equally valid and useful to managing them: the struggle to differentiate oneself from one’s pain, suffering, or illness, and the identification with one’s own pain, suffering or illness [11]. One of Stonington’s patients surprised him by saying, “I want to be here for this, even for the pain. Not really being here would make me suffer” [43]. The pain of childbirth has been claimed by women as an element of self-construction for their own identities as mothers and women in the sense that they wish to be the ones in control of the technology used to alleviate pain, and not to be controlled by such technology [19]. Attitudes like choosing pain or accepting suffering can be a way of affirming the self. For Viktor Frankl [44], accepting unavoidable suffering can even be a way of finding a sense in life; suffering and facing suffering bravely can be a way of affirming one’s own identity, an achievement, a noble cause, instead of a degradation of the self. Suffering can in the end be considered a characteristic of one’s own identity; after so much suffering, the poet Rosalía de Castro finds in herself an empty space that cannot be filled with anything but suffering:

“That at the bottom, the very bottom / of my insides / there is a desert wasteland / unfillable with laughter / or contentment / but with the bitter / fruits of pain!”^11^ [45].

It may be possible to “feel at home in suffering” – not in a masochistic sense, but as a way of dealing with it. As an alternative to the essential definitions, I propose to understand suffering as an unpleasant or even anguishing experience which can severely affect a person on a psychophysical and even existential level.

### Conceptualizing suffering and pain

Conceptualizing suffering as an experience emphasizes the fact that it is something a person experiences (both what Dilthey calls a “lived experience” (Erlebnis), an immediate, unreflected experience and an “ordinary, articulated experience” (Lebenserfahrung) [46, 47]. We should not look at suffering as an abstract phenomenon, but as something experienced by somebody.

Suffering, like pain, is unpleasant or even anguishing: Even if we do not accept an essentialist definition and we reject the understanding of suffering as a “loss of the self” or as a “reaffirmation of the self”, a definition is still necessary. “Unpleasantness” defines suffering and pain. Leknes and Bastian [48] propose “to move beyond a view of pain as simply unpleasant” because “it can also be experienced as pleasant, produce pleasant experiences or motivate us towards pleasant experiences”. They offer a number of advantages and benefits of pain: it represents a possibility for redemption after a transgression, it can highlight bravery, motivate us, enhance sensation, offer temporary relief from other pain and offer “an effective contrast to many non-painful experiences, which can appear relatively pleasant if they occur after pain has ended.” However, such benefits or advantages exist only because pain is unpleasant (if it were not, it would no serve as a redemption, etc). The only convincing argument against the “unpleasantness” of pain is the “pain asymbolia” condition where patients feel pain but not unpleasantness. As I already mentioned, pain consists of a somatosensorial perception followed by a transitory mental image of the local change in the body (nociception) on the one hand, and an unpleasant emotion on the other hand. For Leknes and Bastian, a condition like “pain asymbolia” proves that pain is not necessarily unpleasant. However, I argue that people suffering from such a condition do not have a complete experience of pain, but only of one of its parts. In any case, pain asymbolia is a medical condition rather than a usual experience of pain.^12^

Suffering is not always extreme. Sometimes it is a bearable, short, inconsequential experience. However, it is important to include in our definition the possibility that suffering can affect us at an existential dimension, meaning that it can have an impact on crucial matters regarding one’s personal life, matters that affect our existence in the world, like the desire to continue living, the decision of whether or not to have children, or even how to live life – choices that have to be seen in the context of our attachment to the world. This possibility indeed characterizes suffering too and helps us to perceive its (possible) relevance in life. Moreover, the inclusion of the existential dimension of suffering emphasizes the individual’s capacity for dealing with their unpleasant circumstances/experiences, as well as the crucial impact of their attitude and choices on the whole experience of suffering.

## Discussion

Naturalistic and essential conceptualizations of pain and suffering are not adequate because they can have undesirable epistemological, ontological and moral consequences. The naturalistic approach of classical evidence-based medicine incorporates a particular view of human beings based on the Cartesian mind/body dichotomy, in which the body is understood as a mechanism that works according to universalizeable, manipulable processes. Even if the “humanistic turn” in medicine has started to vindicate more holistic views of the human being, medicine and its disciplines still depend on the idea that the different parts of the body can be treated independently. Moreover, symbolic, subjective and meaningful dimensions of pain and suffering are still not sufficiently taken into consideration. Negative epistemological and practical consequences of such an approach are the impossibility or difficulty of identifying and managing these dimensions of pain and suffering, the fact that unrecognized pain and suffering are inflicted to further particular goals (healing, information gain, prevention), as well as the lack of consideration of concrete phenomena like chronic pain, non-somatic pain or the placebo effect.

Cassell’s medical humanism tries to respond to these problems of classical evidence-based medicine and offers a good conceptualization of pain, concurrent with the results of neurological, sociological and anthropological studies. However, this article criticizes Cassell’s definition of suffering because, despite the fact that it is able to overcome the mind/body dualism, his idea of personhood is still inadequate. The idea that suffering threatens the integrity of a person entails an idea of the person as an autonomous, rational, coherent and well-equilibrated human being – a view which has been rejected by psychological, philosophical and sociological theories in the twentieth century – and an essential definition of suffering. Cassell’s conception of the person can also not be sustained with the help of narrative theories of the self, because the way in which stories concern the construction of personal identity and the way in which they are incorporated into our understanding of our own lives and the lives of others do not support an idea of wholeness; rather, the stories we tell ourselves are always partial, fragmentary and never definitive. Moreover, the fact that suffering can contribute to the creation of identity instead of its destruction contradicts Cassell’s definition.

Phenomenology has contributed to “embodying the mind” and “minding the body” by emphasizing the crucial role of the body in our experience, as can be seen in the explanation of the placebo effect, according to which the body is able to respond meaningfully to a demanding situation (even if we are not conscious of it). However, some phenomenological definitions of suffering (for example, "suffering as an alienation of the self", "suffering as unhomelike being in the world") may suggest essential and universal characteristics of suffering, thus excluding from it other unpleasant or anguishing experiences that the affected themselves indeed consider suffering. A more open definition should be able to incorporate the subjective dimension of suffering, and even the difficulties or impossibility of expressing very extreme experiences, the fact that a person may be suffering without knowing why, or even that he/she may be partially or totally unaware of his/her suffering. Such dimensions of suffering follow from the fact that human beings have irrational and incoherent dimensions which are not transparent to themselves. A person is the ever-changing result of his/her daily struggles, including his/her management of suffering and pain. We have to focus not only on what we “lose” when we suffer, but also on the various cultural, personal and social adaptations and resources to manage suffering.

## Conclusion

Defining suffering substantively turns it into a normative concept, which results in epistemological mistakes and moral injustices. Not all suffering is alienating and it is unfair to deny the suffering of others; for instance, the categorical affirmation that childbirth pain does not entail suffering, as stated by Svenaeus [24], can be unfair. At the same time, not all aspects of suffering can be objectified.

A definition of pain cannot be based only on the neurological understanding of it, but has to incorporate other relevant factors such as cognitive awareness, interpretation, behavioral dispositions, as well as cultural and educational factors beyond the medical sphere. Hence, a formal, non-essential and non-naturalistic conceptualization of both terms is proposed. Suffering is an unpleasant or even anguishing experience which can severely affect a person on a psychophysical and even existential level. Like suffering, pain is also unpleasant. Both are experiences which affect the whole person (not merely their “body” or “mind”), and a crucial aspect of them is the personal attitude and choices which are in turn influenced by cultural and social patterns. Not only the natural sciences, but also the social sciences and humanities play a crucial role in understanding all the dimensions of these phenomena. Additionally, the view of a person as a psychophysical instead of a dualistic being demands a total paradigm shift in medicine and new research approaches which are able to challenge the boundaries of various disciplines.

## Footnotes

1 “Pain tolerance” is defined as “the maximum intensity of a pain-producing stimulus that a subject is willing to accept in a given situation” and “pain threshold” as “the minimum intensity of a stimulus that is perceived as painful” by the International Association for the Study of Pain (http://www.iasp-pain.org/Education/Content.aspx?ItemNumber=1698#Nociception, consulted on 10.02.2016).

2 “Minding the Body” is the title of a paper by Antonio and Hannah Damasio [49].

3 The hedonistic and materialistic Epicurus argued in the fourth century BC for the human being as an entirely material entity. Although he distinguished between the body (*sarx*) and mind (*psyché*), he did not consider them to be different ontological substances (as Descartes did), arguing that they were simply made up of different kinds of atoms. For Epicurus, *sarx* and *psyché* are two parts of a single, whole organism [50], and the mind cannot exist without the body. This distinction parallels the Epicurean distinction between the “pains of the body” (*ponos*) and the “sufferings of the soul” (*lype*). Their opposites are *aponía* (absence of physical pain) and *ataraxía* (absence of spiritual suffering). Total happiness (*eudaimonía*) is possible only when we enjoy both *aponía* and *ataraxía*.

4 I use the term “disease” in the sense of the medically diagnosed pathology, leaving the term “illness” for the subjective experience of the disease by the ill person [27].

5 Van Hooft [26] suggests that Cassell maintains such a distinction; however, this is not the case [25].

6 Not only phenomenological theories have developed alternative conceptualizations of the body. See for example Schicktanz [51] on the different conceptualizations of embodiment in bioethics and their corresponding interpretations of autonomy.

7 According to Leder [32], the body has a tendency of self-concealment, of performing its normal processes and functions without them being “present” for us (i.e. they – and the body in general – are essentially “absent”). However, when we feel pain, the body is no longer “absent”; instead, it is vividly perceived, “present”.

8 Previous interest in psychogenic pain in the history of medicine has been reported, at least since the second half of nineteenth century, as it can be seen in the work of Otto Binswanger [15]

9 For Kuhn, a “paradigm shift” occurs during a scientific revolution. A scientific paradigm is defined as a constellation of facts and theories (assuming that the theories are not exactly developed in order to explain previously given facts, but that facts emerge together with the theories explaining them). In other words, a scientific paradigm includes its own scientific problems, instruments and criteria for solving them, a whole view (Gestalt) of the world.

10 My translation.

11 My translation. “¡Que. no fondo ben fondo / das entrañas / hai un deserto páramo / que non se enche con risas / nin contentos, / senón con froitos do dolor / amargos!”

12 Grahek [52] distinguishes between pain asymbolia and other circumstances in which the painful stimulus seems to be felt without the subsequent unpleasant emotion, like for example lobotomized, cingulotomized and morphinized patients. Only in the case of pain asymbolia do persons not fell the painful stimuli. The other conditions may include cases of indifference to the painful stimuli, but in order to be indifferent to pain, one needs to be able to feel it.
